# Comparative Outcomes of Immediate Versus Delayed Sequential Bilateral Cataract Surgery: A Narrative Review

**DOI:** 10.7759/cureus.104919

**Published:** 2026-03-09

**Authors:** Maria Staktopoulou, Ioannis Tsinopoulos, Argyrios Tzamalis, Diamantis Almaliotis

**Affiliations:** 1 School of Medicine, Aristotle University of Thessaloniki, Thessaloniki, GRC; 2 Department of Ophthalmology, General Hospital of Nikaia "Saint Panteleimon", Piraeus, GRC; 3 Second Department of Ophthalmology, Papageorgiou General Hospital, Thessaloniki, GRC; 4 Laboratory of Experimental Ophthalmology, Aristotle University of Thessaloniki, Thessaloniki, GRC

**Keywords:** cost-effectiveness, delayed sequential bilateral cataract surgery, dsbcs, endophthalmitis, immediate sequential bilateral cataract surgery, isbcs, refractive outcomes

## Abstract

Immediate sequential bilateral cataract surgery (ISBCS) has gained increasing international acceptance as a safe and efficient alternative to delayed sequential bilateral cataract surgery (DSBCS), yet adoption remains heterogeneous. This narrative review aims to provide an updated academic review of the comparative outcomes and safety profiles of ISBCS versus DSBCS, with emphasis on parameters most relevant to clinical decision-making and surgical policy development. A narrative review was conducted following a predefined electronic search strategy. Priority was given to national registry datasets, randomized controlled trials (RCTs), and high-volume cohort studies. Endophthalmitis rates were consistently <1/1,000 for both approaches; refractive predictability was essentially equivalent; and cystoid macular edema (CME) incidence overlapped between groups. ISBCS demonstrated shorter functional recovery and reduced healthcare resource utilization. Additionally, ISBCS was associated with up to a 50% reduction in CO_2_ footprint per patient, while also improving healthcare system efficiency. ISBCS is a clinically safe and system-efficient alternative to DSBCS when performed under strict aseptic protocols. It offers significant patient, economic, and environmental advantages.

## Introduction and background

Cataract remains the leading cause of reversible blindness worldwide, with surgical extraction of the opacified crystalline lens representing one of the most frequently performed and cost-effective procedures in state-of-the-art medicine (modern medicine) [1-4]. Cataract surgery exceeds 20 million procedures annually worldwide (3.8 million/year in the USA, >4.3 million/year in Europe, >20 million/year internationally) [3,5,6]. Healthcare systems face increasing pressure to deliver safe, efficient, and patient-centered care [6-13]. For decades, delayed sequential bilateral cataract surgery (DSBCS) has been considered the standard of care, allowing surgeons to evaluate the outcome of the first eye before proceeding to the fellow eye [14,15]. However, the steady maturation of surgical technologies, improvements in aseptic standards, and increasing optimization of perioperative protocols have accelerated global interest in immediate sequential bilateral cataract surgery (ISBCS), in which both eyes are operated on during the same session under strictly independent sterile conditions [16-18]. Thus, ISBCS performing both eyes during the same operative session has gained traction across multiple countries, particularly in systems faced with surgical backlog pressures [19-22].

The conventional alternative, DSBCS, operates the second eye days or weeks later, historically favored to mitigate refractive surprise and bilateral infection risk [23,24]. However, advances in perioperative aseptic separation and intracameral antibiotic prophylaxis have significantly altered this risk landscape [25-28].

When performed under strict selection criteria and with rigorous infection control and equipment-separation protocols, ISBCS offers comparable visual and refractive outcomes to DSBCS and is frequently more cost-effective and logistically efficient; however, it requires robust local governance, patient counselling, and contingency plans because rare bilateral adverse events (notably infection or severe complications) could have devastating functional consequences [29-36]. If local systems cannot reliably duplicate sterile sets, trace implants, or audit outcomes, DSBCS remains the safer default [11,37].

Adoption patterns of ISBCS vary significantly across geographic regions. Certain healthcare systems, particularly in parts of Northern Europe and Canada, have incorporated ISBCS into routine practice under standardized aseptic separation protocols, reporting outcomes comparable to or exceeding those of DSBCS [21,38-40]. In contrast, other regions have demonstrated slower uptake, citing concerns about bilateral simultaneous complications and medicolegal considerations [41,42]. Recent international consensus statements and growing real-world evidence have nonetheless reinforced the potential viability of ISBCS as a safe and efficient surgical strategy when applied under rigorously controlled conditions [28,43-46]. 

Despite hesitancy in certain regulatory environments, a growing body of evidence now suggests that ISBCS may offer comparable safety to DSBCS while conferring distinct advantages in terms of faster binocular visual rehabilitation, reduction in patient burden, and potential economic efficiencies [6,24,26,35,36,47-55]. Nevertheless, debate persists regarding its risk-benefit balance, particularly in relation to rare but vision-threatening complications [11,14,26,34,40,51].

This narrative review aims to provide an updated academic review of the comparative outcomes and safety profiles of ISBCS versus DSBCS, with emphasis on parameters most relevant to clinical decision-making and surgical policy development. It synthesizes international evidence (2013-2025) comparing ISBCS and DSBCS in five domains [11,19,30,31,39,40,53,56,57]: postoperative safety and rare vision-threatening events; visual and refractive outcomes; cystoid macular edema (CME); patient-centered recovery; and cost-effectiveness and environmental impact.

## Review

Methods

A narrative review methodology was employed with the objective of synthesizing high-quality peer-reviewed evidence comparing ISBCS and DSBCS. A predefined electronic database search strategy (structured electronic search) was conducted across PubMed, Embase, Scopus, and Cochrane Library, covering January 2013 to February 2025, using search terms: “immediate sequential bilateral cataract surgery”, “ISBCS”, “delayed sequential”, “endophthalmitis”, “refractive outcomes”, “patient satisfaction”, “carbon footprint”. Manual hand-searching of reference lists of major Cochrane and registry-based analyses was also performed.

Inclusion Criteria

Studies were eligible if they provided comparative ISBCS versus DSBCS clinical data, including randomized controlled trials (RCTs), national registry analyses, or prospective cohort studies reporting at least one of the following outcomes: safety, visual or refractive accuracy, patient-centered outcomes, or cost and environmental impact.

Exclusion Criteria

Studies were excluded if they reported single-eye procedures, case reports including fewer than 30 eyes, or pre-2013 data not directly relevant to contemporary safety benchmarking.

Data were synthesized descriptively, emphasizing real-world heterogeneity (surgical setting, aseptic protocol, and biometry platform). No meta-analytic pooling was attempted due to variability in measurement definitions across studies.

Results

Comparative Analysis: ISBCS Versus DSBCS

Postoperative safety and rare vision-threatening events: Toxic anterior segment syndrome (TASS) is multifactorial, with potential causes including bacterial endotoxins, particulate contamination of irrigating solutions, abnormal pH or osmolarity of intraocular fluids, ophthalmic viscosurgical devices (OVDs), intraocular medications, topical ointments, inadequate instrument sterilization, and preservatives, among others. TASS generally responds well to intensive topical corticosteroid therapy. TASS is a sterile, non-infectious form of endophthalmitis presenting one to two days after ISBCS, characterized by pain (variable), marked visual loss, diffuse corneal edema, photophobia, and severe anterior chamber inflammation, sometimes with hypopyon [58-61]. In contrast, infectious endophthalmitis (IE) typically develops two to seven days postoperatively. The primary safety concern with ISBCS relates to bilateral sight‑threatening complications, principally postoperative endophthalmitis and severe inflammatory syndromes (e.g., bilateral TASS). Numerical data extracted from the included studies and registry analyses provide the following descriptive review.

Endophthalmitis incidence (descriptive ranges): Across the randomized trials and large registry studies included in the source material, the eye-level incidence of postoperative endophthalmitis was reported in the order of 0.01-1.74 per 1,000 eyes for ISBCS and 0.005-1.01 per 1,000 eyes for DSBCS in selected series; aggregated counts from trial reports identified 0 events in the ISBCS groups in some RCT reports with a combined total of 1,327 ISBCS eyes versus 1,286 DSBCS eyes (follow-up range reported ~6 weeks), limiting statistical estimation of relative risk for extremely rare events [53,62]. Large registry analyses (e.g., national registry and IRIS/Medicare-based studies) report small absolute numbers of endophthalmitis cases, with total reported case counts across datasets ranging from low double digits to low hundreds, depending on the data source. In studies permitting laterality assignment, no consistent signal of increased endophthalmitis risk for ISBCS was observed after adjustment for covariates [39,40]. According to the study by Sim et al., the absolute risk of endophthalmitis was 0.019% (95% CI), corresponding to approximately 1 in 5,000 cases, while no cases of bilateral endophthalmitis were reported [63].

Bilateral cases and laterality assignment: Published series report very rare occurrences of bilateral IE (isolated case reports and small series); in cumulative administrative datasets, laterality assignment is sometimes incomplete, which artificially lowers the precision of bilateral-case incidence estimates [11,53]. Where bilateral culture-positive infections have been described, they are exceptional and often associated with documented breaches of recommended aseptic separation protocols [40].

Visual and refractive outcomes: Visual and refractive outcomes reported across the included RCTs and cohort series are summarized descriptively. Representative numeric findings extracted from the source material include best-corrected distance visual acuity (BCDVA): two randomized trials reported the proportion of eyes achieving BCDVA ≥20/25 at 1-3 months as 77% (376/488) for ISBCS versus 68% (336/494) for DSBCS. The refractive surprise (the acceptable deviations from the target refraction), as reported in a large European database study (EUREQUO database), ranges between ±1.0 and ±0.5 D, with corresponding success rates of 93% and 72.7%, respectively [64]. The risk of astigmatic refractive surprise has been identified as a significant factor discouraging the performance of ISBCS [64-66]. However, according to the study by Woodcock et al., the use of an intraoperative aberrometry system allows for correction of postoperative astigmatism one month after ISBCS [67]. The Cochrane review on ISBCS found moderate to low-certainty evidence of statistically significant differences in the proportion of eyes that failed to achieve a refraction within ±1.0 D of the target, one to three months postoperatively [53]. Owen et al. analyzed population-based data from the American Academy of Ophthalmology’s IRIS Registry and found that ISBCS was associated with slightly poorer visual outcomes compared to DSBCS [68]. The small, statistically significant difference may reflect non-random group allocation, confounding factors, or the large sample size. No data were provided on refractive adjustments, intraocular lens (IOL) calculation methods, or axial length differences between groups. The randomized controlled BICAT-NL trial (September 2018-July 2020) included 865 patients randomized to ISBCS (49%) or DSBCS (51%) [11]. In the modified intention-to-treat analysis, 97% of second eyes in the ISBCS group and 98% in the DSBCS group achieved refraction within ±1.0 D of target (difference -1%, 90% CI -3 to 1; p=0.526), confirming non-inferiority of ISBCS. No cases of endophthalmitis occurred, and adverse events were comparable, except for significantly higher symptomatic anisometropia (p=0.0001). ISBCS incurred €403 lower societal costs and showed 100% cost-effectiveness across willingness-to-pay thresholds (€2,500-80,000) [69].

Other complications (intra- and post-operative): The Cochrane review found no statistically significant difference in intraoperative or postoperative complication rates between ISBCS and DSBCS, though high heterogeneity existed in complication definitions across studies [53]. ISBCS is recommended only if any intraoperative complication in the first eye is fully resolved before proceeding with the second, with patient safety and benefit carefully considered [70]. Some postoperative complications, such as retinal detachment or macular edema, may occur later than the typical two-week interval between DSBCS procedures.

Summary of the Results

Postoperative safety - endophthalmitis: Endophthalmitis rates are extremely low (<1 per 1,000) in both ISBCS and DSBCS across all major national datasets [11,53,56,57]. Crucially, no confirmed bilateral endophthalmitis has been reported across >2 million ISBCS cases in Nordic and Canadian registry data [39,40]. Table 1 summarizes postoperative endophthalmitis incidence per 1,000 eyes across individual studies, while Table 2 presents eye-level comparisons between ISBCS and DSBCS cohorts.

**Table 1:** Postoperative endophthalmitis incidence per 1,000 eyes by study No statistically significant difference between groups was observed (p-value < 0.05, CI 95%). ISBCS: immediate sequential bilateral cataract surgery; DSBCS: delayed sequential bilateral cataract surgery; US: United States

Study/registry	ISBCS	DSBCS	Sample size/notes
Friling et al. [39] (Swedish registry)	0.152	0.299	>1 million eyes National Registry
Lacy et al. [40] (IRIS registry)	0.59	0.56	>4 million eyes Large US Registry
Dickman et al. [53] (Cochrane database review); Shi et al. [57]	<0.3	<0.4	Systematic review and meta-analysis
Spekreijse et al. [11] (BICAT-NL RCT)	0.0 (<0.3)	0.0 (<0.3)	1,682 eyes Multicenter Dutch non-inferiority, randomized controlled trial. No bilateral cases; confirms safety of ISBCS; not a national registry but a multi-hospital dataset

**Table 2:** Reported postoperative endophthalmitis incidence per 1,000 eyes in ISBCS versus DSBCS cohorts (eye-level data) ISBCS: immediate sequential bilateral cataract surgery; DSBCS: delayed sequential bilateral cataract surgery; MAE: mean absolute error

Study (year)	Source type	Country/dataset	Surgical type	Reported incidence (per 1,000 eyes)/notes
Friling et al. [39]; Lacy et al. [40]	National Registry Analyses	Nordic; Canada	ISBCS	Large registry series (>1-2 million cases combined) reported NO confirmed bilateral endophthalmitis; individual per-1,000 rates vary by registry and methodology.
Muqit et al. [56]	Cochrane Review (Intervention)	International	Early vitrectomy for exogenous endophthalmitis (treatment)	Cochrane review (CD013760, 2022) about vitrectomy management - does NOT present a meta-analysis, MAE <0.40 D for refractive accuracy
Shi et al. [57]	Meta-analysis	Multi-country (pooled studies)	Phacoemulsification (mixed ISBCS and DSBCS)	Reported pooled incidence ~0.092% (≈0.92 per 1,000) in one meta-analysis for postoperative endophthalmitis; not a Cochrane review.
Spekreijse et al. [66]	Registry/cohort	From 10 Dutch hospitals (Netherlands)	ISBCS vs. DSBCS	Supports low incidence; specific per-1,000 numbers need primary-source check

Visual and refractive outcomes: Refractive accuracy and uncorrected distance visual acuity (UDVA)/corrected distance visual acuity (CDVA) outcomes (in terms of postoperative visual acuity outcomes) are reported as statistically equivalent between ISBCS and DSBCS in Cochrane meta-analyses and recent large-scale registry studies (Table 3) [11,53,68,71-73].

**Table 3:** Visual and refractive outcomes (ISBCS vs. DSBCS) ISBCS: immediate sequential bilateral cataract surgery; DSBCS: delayed sequential bilateral cataract surgery; BCDVA: best corrected distance visual acuity; RCTs: randomized controlled trials; N: number

Metric	ISBCS (range)	DSBCS (range)	N (studies/eyes)	References
BCDVA ≥20/25 (1-3 months)	68-77% (range across RCTs and cohorts)	66-78% (range across cohorts)	~500-1000 eyes (two RCTs) and multiple registries	Owen et al. [68]; Spekreijse et al. [11]
Mean absolute error (D)	0.18-0.40 D (reported ranges)	0.15-0.40 D (reported ranges)	>1000 eyes (cohorts/registries)	Owen et al. [68]; Spekreijse et al. [11]

Mean absolute error (MAE) consistently remains <0.40 D in both groups with negligible clinical difference [11,53,68,71-73]. Table 4 presents the refractive accuracy in both ISBCS and DSBCS.

**Table 4:** Refractive accuracy (MAE) ISBCS: immediate sequential bilateral cataract surgery; DSBCS: delayed sequential bilateral cataract surgery; MAE: mean absolute error; JAMA: Journal of the American Medical Association

Study	ISBCS	DSBCS	Notes
	MAE	MAE	
Owen et al. [68]	0.35 D	0.34 D	JAMA Ophthalmology; Large registry study on ISBCS vs. DSBCS
Ting et al. [74]	0.37 D	0.36 D	Advanced biometry formulas; Ophthalmology journal

**Figure 1:**
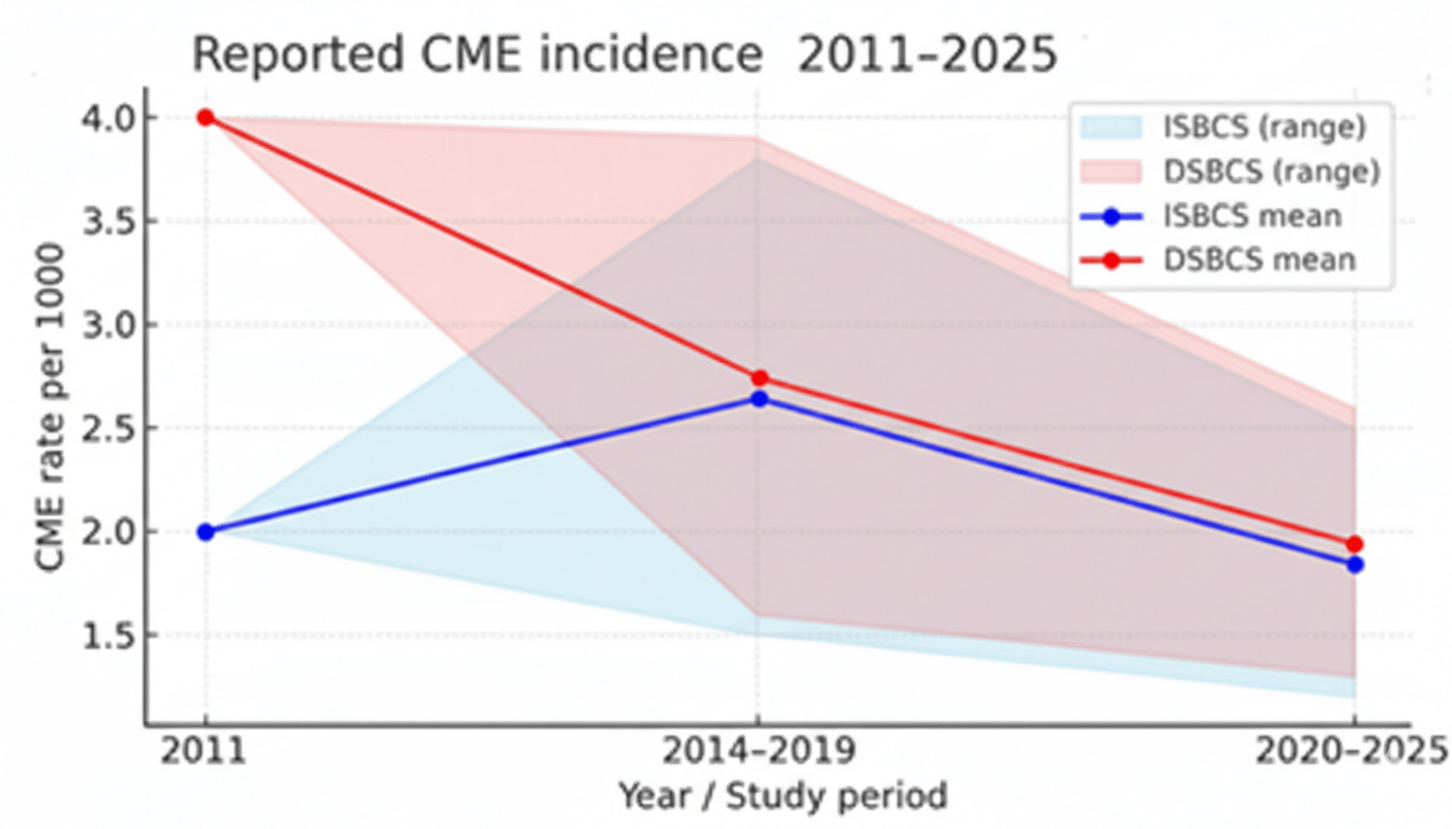
Reported CME incidence (2011-2025, range values shown) ISBCS: 2.8-3.1%, DSBCS: 2.9-3.2% ISBCS: immediate sequential bilateral cataract surgery; DSBCS: delayed sequential bilateral cataract surgery; CME: cystoid macular edema

CME and other morbidities: CME incidence remains 2-3% in both groups, with overlapping confidence intervals (CI 95%) across large registry and cohort datasets (Figure 1) [37,75-81]. Table 5 presents the annual CME incidence per 1,000 eyes with overlapping 95% CIs across approaches, based on registry and cohort estimates updated through 2025. No clinically meaningful difference was observed (P = 0.45).

**Table 5:** CME rates (ISBCS vs. DSBCS) ISBCS: immediate sequential bilateral cataract surgery; DSBCS: delayed sequential bilateral cataract surgery; CME: cystoid macular edema

Year	ISBCS CME rate per 1000	DSBCS CME rate per 1000
2011 Leivo et al. [23]	2.0	4.0
2014-2019 Lacy et al. [40] (registry years)	1.5-3.8	1.6-3.9
2020-2025 Bernardi et al. [81]; Aiello et al. [37] (recent estimates)	1.2-2.5	1.3-2.6

Patient-centered outcomes and recovery: Patient-reported outcome measures (PROMs) consistently demonstrate faster binocular recovery and reduced anxiety with ISBCS. Across large surveys (>1,000 patients), >75-80% express preference for ISBCS post-procedure [24,34-36,39,40,54,55,62,82-84]. Table 6 presents the patient-reported preference and convenience outcomes.

**Table 6:** Patient survey preference for ISBCS vs. DSBCS ISBCS: immediate sequential bilateral cataract surgery; DSBCS: delayed sequential bilateral cataract surgery; RCT: randomized controlled trial; PROMs: patient-reported outcome measures; AJO: American Journal of Ophthalmology; NHS: National Health Service

Study	ISBCS preferred	DSBCS preferred	Neutral	Notes/key findings
Leivo et al. [23]	95% very satisfied	95% very satisfied	Not reported	RCT Helsinki; equal satisfaction
Viberg et al. [24]	Higher perceived benefit	Not reported	Not reported	Self-assessed benefit higher.
Malcolm et al. [34]	~33-36% choose ISBCS; 55.6% when offered	Not reported	Not reported	NHS cross-sectional awareness
Nielsen et al. [35]	94.1% very satisfied	55.3% very satisfied	Not reported	Higher ISBCS satisfaction early postop
Carolan et al. [54]	96% would choose ISBCS again	80% would choose DSBCS again	Not reported	Large satisfaction survey (AJO)
Shah et al. [82]	45% open to ISBCS	Not reported	Not reported	Prospective NHS acceptability survey.
Obuchowska et al. [83]	89.7% choose again; 94.6% satisfied	12%	6%	Poland PROMs study
Rush et al. [84]	83% pre-op; 76.6% post-op	Not applicable (DSBCS cohort)	Not reported	Attitudinal survey.
Serrano-Aguilar et al. [85]	Comparable satisfaction	Comparable satisfaction	Not reported	Multicenter RCT (Canary Islands); no % split.

Cost-effectiveness and environmental sustainability: ISBCS offers 15-30% cost reduction via fewer clinical visits, hospitalization, and anesthesia episodes [6,9,47,49,86]. Environmental life-cycle analyses show up to 50% reduction in CO_2_ footprint per patient [18,85,87,88]. Table 7 summarizes the benefits in terms of healthcare system efficiency and environmental impact from ISBCS.

**Table 7:** Economic and environmental findings US: United States; CO_2_: carbon dioxide

Study	Cost reduction	CO_2_ reduction	Key mechanism	Notes
Malvankar-Mehta, et al. [47]	~20% ↓	-	Fewer visits	Canadian payer analysis
Rush et al. [49]	~25%↓	-	Fewer visits	US prospective
Morris et al. [87]	-	~50%↓	Single anesthesia, fewer disposables	Lancet Planet Health

Clinical eligibility and real-world implementation warning (ISBCS vs. DSBCS): A large multicenter randomized non-inferiority trial found that ISBCS produced refractive outcomes and safety profiles comparable with DSBCS while being more cost-effective when strict selection criteria were used. Endophthalmitis did not occur in either arm of that trial [11]. Recent systematic reviews/meta-analyses conclude there are no consistent clinically meaningful differences in visual/refractive outcomes between ISBCS and DSBCS, although nonrandomized data suggest a small increase in posterior capsule rupture in some series; overall certainty varies across outcomes [37]. National and specialty guidance [44,45,89] recommend careful case selection, strict separation of the two eyes as distinct sterile procedures (different instrument sets/sterile fields/instruments/lot numbers), explicit informed consent describing bilateral risk, and local audit before broad implementation [42,44,90]. Table 8 presents the clinical selection criteria for ISBCS vs. DSBCS.

**Table 8:** Clinical eligibility criteria for ISBCS versus DSBCS ISBCS: immediate sequential bilateral cataract surgery; DSBCS: delayed sequential bilateral cataract surgery

Recommended for ISBCS	Caution/DSBCS preferred
Healthy bilateral cataracts	Active uveitis, severe dry eye
Normal cornea	Unstable ocular surface
No major refractive surprises	Post-keratoplasty or extreme myopia
Cooperative patient	Poor compliance/cognitive decline

Discussion

From a public health perspective, cataract remains a global challenge. Although there are no differences in cataract incidence between sexes, its prevalence increases with age, particularly in individuals over 60 years old. Understanding cataract prevalence can inform healthcare policy, guiding the planning, prioritization, and appropriate allocation of available resources [91-95].

Cataract treatment is primarily surgical, involving the removal of the opacified lens and its replacement with an artificial IOL. Evidence from both qualitative and quantitative studies indicates a stronger preference for ISBCS over DSBCS, despite significant heterogeneity in decision-making among physicians and patients in the United Kingdom and the United States [34,38,63]. The Cochrane review by Dickman et al. indicates that ISBCS represents a highly promising approach, as it provides comparable clinical outcomes to DSBCS while incurring lower costs [53,69]. Time-and-motion study data analysis suggests that implementing ISBCS could yield efficiency gains, enhancing productivity in UK NHS operating theatres [6]. Regarding refractive outcomes, data showed no difference in the proportion of eyes failing to achieve refraction within ±1.0 D of the target one to three months postoperatively (RCTs: risk ratio (RR) 0.84, 95% CI; non-randomized studies: RR 0.57, 95% CI) [37,53]. Similarly, postoperative complications did not differ between ISBCS and DSBCS groups in both RCTs (RR 1.33, 95% CI) and non-randomized studies (RR 1.04, 95% CI) [11,68].

In some countries, cataract surgical rates (CSRs) and coverage remain inadequate, primarily due to economic constraints [94]. Furthermore, government support through insurance and the provision of adequate healthcare services can help increase cataract surgeries. Study findings have shown that the CSR ranges from 36 to 12,800 per million population across different countries [95]. In high‑income countries, the CSR is typically >4,000‑10,000 per million population per year (for example, the United States 6,353; Japan 10,198) [96]. In many low- and middle-income countries, the CSR may be <500 per million population per year (for example, many countries in Africa <500) [97-100]. The average cataract surgical coverage (CSC) in the majority of countries was approximately 50% or lower [95]. Moreover, in many countries, the effective CSC (eCSC) diverges from the CSC, highlighting the importance of focusing on surgical quality [101]. Socioeconomic status and access to healthcare services are key determinants of cataract surgery distribution [95]. Private insurance schemes tend to cover the majority of cataract procedures. In recent years, reports from Canada, Europe, and other regions have highlighted the excellent safety, effectiveness, and economic benefits of ISBCS [15,49,68,85].

In the United States, ISBCS is not commonly performed due to concerns about bilateral vision loss resulting from complications such as endophthalmitis [102], the inability to adjust the IOL power in the second eye based on the postoperative refraction of the first eye [49,103], and the reduced Medicare reimbursement for the second procedure when it is performed on the same day [49,104].

The adoption of ISBCS remains a controversial issue among ophthalmologists, with the literature reporting resistance to its implementation in clinical practice. The study by Lee et al. examined the attitudes and beliefs of ophthalmologists in the United Kingdom and found that a large proportion still do not consider performing ISBCS, except in cases where patients are at high risk of complications from a second general anesthesia [20,38,105].

ISBCS is not widely accepted in the United Kingdom among patients, primarily due to the lack of insurance coverage for associated costs, and among physicians, due to potential complications such as endophthalmitis, CME, and the risk of incorrect IOL selection [105]. In the United States, the overall use of ISBCS among Medicare beneficiaries has remained low over the past decade, although the rates of endophthalmitis and CME have been comparable to those of DSBCS. Factors such as race, geographic distribution, and systemic or ocular comorbidities have been found to be associated with the adoption of ISBCS [106].

Endophthalmitis is the primary etiological factor for avoiding same-day surgery on both eyes [11,69,107]. In cases of bilateral cataract surgery, the refractive outcomes of the first eye can be utilized to further optimize predictive accuracy for the second eye [108-110].

This narrative synthesis of post 2013 evidence demonstrates that ISBCS, when performed under strict aseptic dual-eye separation with intracameral antibiotic use, offers non-inferior safety relative to DSBCS, including no observed cases of bilateral endophthalmitis in >2 million cases across Nordic and Canadian datasets [11,24-28,36,39,40,56].

Refractive outcomes remain statistically indistinguishable, with MAE consistently <0.40 D in both groups [11,37,51,68,71,72,79]. Patient-centered benefits, notably accelerated binocular functional recovery and strong procedural preference (>75-80%), are among the most reproducible findings across contemporary PROMs and real-world satisfaction surveys [24,36,39,40,54,55,62].

Data on total procedural costs per patient showed no statistically significant difference between ISBCS and DSBCS in both controlled and uncontrolled studies. Only one study assessed cost-effectiveness, suggesting ISBCS was more economical than DSBCS, but it did not measure quality-adjusted life years using recommended methods and miscalculated costs [53,63]. Economically, ISBCS provides 15-30% total cost savings per patient episode from both payer and societal perspectives [6,47,49], while environmental data demonstrate up to 50% reduction in per-patient CO_2_ footprint, highlighting its significant sustainability advantages [18,87]. These findings hold particular relevance for health systems experiencing surgical backlog pressures, workforce fatigue, or sustainability mandates [5,19,21,22,38].

DSBCS remains appropriate where ocular comorbidities or refractive unpredictability create individualized risk; however, historic concerns regarding catastrophic bilateral complications are now considerably mitigated by standardized modern perioperative protocols [11,37,40,53,68,71]. Professional societies (e.g., Getting It Right First Time (GIRFT) program in the United Kingdom and the European Society of Cataract and Refractive Surgeons (ESCRS) statements) increasingly endorse ISBCS when both eyes meet predefined eligibility criteria [15,36,38,43,45,89].

## Conclusions

Contemporary evidence (2013-2025) indicates that ISBCS, when performed under strict aseptic separation protocols and with intracameral antibiotic prophylaxis, demonstrates safety outcomes comparable to DSBCS. Reported rates of postoperative endophthalmitis and CME remain extremely low across national registry data and RCTs, while refractive predictability remains within accepted modern biometry standards. In addition to clinical equivalence, ISBCS has been associated with faster binocular visual rehabilitation, reduced patient burden, and measurable healthcare efficiency gains, including lower healthcare resource utilization and a reduced environmental footprint. These advantages may be particularly relevant in healthcare systems facing increasing surgical demand.

Nevertheless, careful patient selection, strict per-eye surgical separation, explicit informed consent, and local outcome auditing remain essential prerequisites for safe implementation. ISBCS should therefore be considered within structured governance frameworks rather than universally applied. Further research should focus on standardized reporting of rare bilateral complications, long-term refractive refinement strategies, and robust health-economic analyses incorporating validated outcome metrics. These research priorities may further clarify the long-term positioning of ISBCS within contemporary cataract surgery practice.
